# Prognostic Impact of Minimal Pelvic Fluid in Locally Advanced Pancreatic Cancer: A Multicenter Retrospective Study

**DOI:** 10.5152/tjg.2023.23309

**Published:** 2023-12-01

**Authors:** Jungnam Lee, Jin-Seok Park, Seok Jeong, Don Haeng Lee, Jung-Hyun Lim, Soon Gu Cho, Chang Il Kwon, Jong Jin Hyun, Jung Wan Choe, Jae Hee Cho, Sung Ill Jang

**Affiliations:** 1 Division of Gastroenterology, Department of Internal Medicine, Inha University Hospital, Inha University College of Medicine, Incheon, Republic of Korea; 2 National Center of Efficacy Evaluation for the Development of Health Products Targeting Digestive Disorders (NCEED), Inha University Hospital, Incheon, Republic of Korea; 3 Utah-Inha DDS & Advanced Therapeutics Research Center, Incheon, Republic of Korea; 4 Department of Radiology, Inha University Hospital, Inha University College of Medicine, Incheon, Republic of Korea; 5 Department of Internal Medicine, Digestive Disease Center, CHA Bundang Medical Center, CHA University, Seongnam, Republic of Korea; 6 Department of Internal Medicine, Korea University Ansan Hospital, Ansan, Republic of Korea; 7 Department of Internal Medicine, Digestive Disease Center, Gangnam Severance Hospital, Yonsei University College of Medicine, Seoul, Republic of Korea

**Keywords:** Pancreatic neoplasms, computed tomography, survival, minimal pelvic fluid

## Abstract

**Background/Aims:**

Minimal pelvic fluid (MPF) is occasionally encountered on computed tomography (CT) scans during the initial staging of newly diagnosed pancreatic cancer. However, its clinical relevance has scarcely been studied. This study intends to explore the incidence of minimal pelvic fluid and its relevance in terms of survival in locally advanced pancreatic cancer (LAPC) patients.

**Materials and Methods:**

The medical records of patients with LAPC at 4 tertiary referral institutions were retrospectively reviewed from January 2005 to December 2015. Minimal pelvic fluid was defined as a fluid collection volume in the pelvic cavity of <100 mL as determined by abdominal CT. The association between the presence of MPF and patient survival was evaluated.

**Results:**

A total of 59 patients (male:female, 33:26; median age, 68 years; range 46-82 years) with LAPC were enrolled. Of the 59 patients, 22.0% (n = 13) had MPF, and 78.0% (n = 46) had no pelvic fluid (NPF). Baseline clinical characteristics in the 2 groups, including extent of the tumor stage, extent of spread to the lymph nodes stage, and pattern of treatments, were not significantly different. However, median overall survival was significantly less in the MPF group [9.7 months, (95% CI, 5.9-13.5)] than in the NPF group as determined by the log-rank test [16.9 months, (95% CI, 9.3-24.5)] (*P* = .002), and univariate and multivariate analyses showed that the presence of MPF independently predicted a poor prognosis.

**Conclusion:**

The presence of MPF was found to be significantly associated with reduced survival and an independent poor prognostic biomarker in LAPC patients.

**Main Points**

- Minimal pelvic fluid (MPF), which is a small volume of fluid found in the pelvic area, was found to be an indicator of poor prognosis in patients with locally advanced pancreatic cancer (LAPC). The presence of MPF was associated with a significantly shorter median overall survival (OS) compared to the no pelvic fluid (NPF) group.
- The MPF group was associated with a median OS of 9.7 months, while the NPF group had a median OS of 16.9 months, indicating a substantial difference in survival rates.
- The results suggest that patients with MPF should be considered equivalent to stage IV metastatic pancreatic cancer and treated accordingly. One of the potential mechanisms for the association of MPF with a worse prognosis could be that it serves as an indirect marker of peritoneal carcinomatosis.
- The study underscores the need for initial stage workups in patients with newly diagnosed pancreatic cancer to include the pelvic area, as this could have implications on treatment decisions and accurate staging of LAPC. The MPF should not be disregarded as physiological fluid due to its significant association with poor prognosis.

## Introduction

Pancreatic cancer ranks as the seventh major cause of cancer death and is projected to become the third leading cause by 2025.^1, 2^ Despite decades of effort to improve the diagnosis and treatment of pancreatic cancer, over 80% of cases are diagnosed with locally advanced or metastatic disease, making them ineligible for curative treatment.^3^ Only 20% of localized cases are suitable for curative treatment through surgical resection and adjuvant therapy, yet 80% of these will recur.^3, 4^ Thirty percent of cases have locally advanced pancreatic cancer (LAPC), which is surgically unresectable but confined to the pancreas without distant metastases.^5, 6^

Treatment options include chemotherapy, radiation therapy, and surgery.^7^ The management and outcome of LAPC have significantly improved with the introduction of new chemotherapy regimens.^8, 9^ These regimens have demonstrated favorable results, with stable disease or tumor regression observed in most of patients after 4-6 months of induction chemotherapy.^8, 9^ Remarkably, 12%-35% of these patients achieve successful downstaging to resectable disease.^5^ Therefore, accurate staging, using techniques such as computed tomography (CT) scans, is vital for treatment planning. Computed tomography scans are recognized as the optimal initial imaging modality, providing a detailed evaluation and insights into treatment strategies for pancreatic cancer, and high-resolution CT has become the cornerstone in determining resectability.^7^

Malignant ascites, characterized by the presence of malignant cells in the peritoneal cavity, is closely associated with peritoneal carcinomatosis.^10^ About 10% of all ascites cases are malignantly related, with a particularly grim prognosis for patients with gastrointestinal cancer.^11, 12^ Interpretation of ascites can be ambiguous, and the significance of small peritoneal fluid accumulation remains unclear, especially in men or postmenopausal women.^13, 14^ Ascites is also present in various noncancerous diseases and conditions causing fluid retention.^15, 16^ Minimal pelvic fluid (MPF) is sometimes detected during the initial staging of gastrointestinal cancers, but few studies have addressed its clinical relevance. Interestingly, in lung cancer, the presence of minimal pleural effusion is an essential poor prognostic factor, especially in early-stage cancer.^17^

In pancreatic ductal adenocarcinoma (PDAC), ascites detection frequently comes late, with grim outcomes, as the median survival duration after ascites onset is 1.8 months.^10^ Ascites management focuses on symptom relief and underlying malignancy treatment. Although patients with malignant ascites have a limited life expectancy, there is a lack of substantial data on the clinical importance of ascites when it develops early on at presentation or when it occurs in patients with localized nonmetastatic disease. Clinical decisions are challenging when patients have small amounts of radiographic ascites without other metastases, particularly regarding the appropriateness of local therapies such as surgery or radiotherapy. The precise incidence and clinical relevance of MPF in pancreatic cancer remain unexplored. This study aims to investigate the incidence of MPF and its prognostic role in LAPC, potentially guiding future therapeutic options for these patients. 

## Materials and Methods 

### Patients

In this retrospective study, we conducted a thorough examination of the medical records from 4 reputable tertiary referral institutions (Inha University Hospital, CHA Bundang Medical Center, Korea University Ansan Hospital, and Gangnam Severance Hospital). Our objective was to investigate LAPC cases diagnosed between January 2005 and December 2015. This extensive study duration allowed us to accumulate a substantial dataset that would enable meaningful analysis. To ensure the reliability and accuracy of our findings, we implemented stringent inclusion and exclusion criteria. Eligible patients were diagnosed with LAPC according to the eighth edition of the American Joint Committee on Cancer (AJCC) staging system, specifically meeting the criteria of stage III pancreatic cancer (T1, T2, or T3 with N1M0 staging or T4 with any N stage and M0 staging). Furthermore, patients needed to have a confirmed diagnosis of PDAC, supported by pathological evidence. Additionally, their medical records needed to include available abdominal CT images including the pelvic cavity at the time of diagnosis. To specifically investigate the impact of nonsurgical treatment approaches, patients who underwent surgical intervention at the time of diagnosis were excluded from our study or subsequent analyses. However, patients who initially received upfront treatment and later required additional surgery after disease control were included in the study or analysis, allowing us to capture the comprehensive spectrum of treatment modalities and their outcomes.

To maintain the integrity of the study and ensure a focused analysis, we excluded cases lacking histological confirmation and patients with specific conditions that could potentially impact the presence of ascites or pelvic fluid. These exclusions encompassed individuals with double primary cancer, underlying liver cirrhosis, end-stage renal disease, chronic heart failure, or hypoalbuminemia (<2.8 g/dL). We also excluded females of childbearing age and patients who were lost to follow-up within 3 months after diagnosis. By employing these stringent criteria, we aimed to gather a well-defined cohort of patients with LAPC, ensuring the reliability and relevance of our study findings.

The study protocol was submitted to and reviewed by the Institutional Review Board (IRB) of Inha University Hospital. After careful evaluation, the study was granted approval with the assigned IRB number 2021-08-028. All aspects of the study, including data collection, analysis, and publication, were conducted in compliance with the ethical principles outlined in the Declaration of Helsinki.

In the study, a thorough examination of their clinical information and demographics was conducted by extracting relevant data from the medical records. The collected data encompassed various factors, including age, sex, and the presence of comorbidities such as diabetes mellitus (DM) and hypertension, which are known to potentially influence the prognosis and treatment outcomes in pancreatic cancer patients.

Furthermore, we diligently documented specific information related to the treatment received by each patient. This encompassed details regarding the chemotherapy regimens and radiation therapy are administered as part of their management. Additionally, we recorded important biomarkers indicative of disease progression and response to treatment, such as levels of carbohydrate antigen 19-9 (CA 19-9), a widely recognized tumor marker, at the time of initial diagnosis. C-reactive protein (CRP) levels, primary tumor size, and the presence or absence of lymph node metastasis were also analyzed. To accurately classify and assess the extent of disease, clinical staging was performed in accordance with the eighth edition of the AJCC guidelines during the initial evaluations. Specifically, for patients with locally advanced unresectable pancreatic cancer, certain criteria were applied to determine their eligibility for inclusion in the study. These criteria included the presence of arterial encasement, defined as the involvement of more than 180° of the superior mesenteric artery or celiac axis by the tumor, as well as the identification of an unreconstructible superior mesenteric vein/portal vein due to tumor involvement or occlusion.

## Definition of Minimal Pelvic Fluid

In our study, MPF was defined based on the assessment of free fluid collection volume within the pelvic cavity, specifically focusing on a volume of less than 100 mL in the pouch of Douglas, as determined through careful analysis of abdominal CT scans. To ensure accuracy and consistency in the interpretation of imaging data, all scans were reviewed by a board-certified radiologist (S.G. Cho.) The presence of pelvic fluid was defined based on radiologic criteria, specifically the identification of a reasonably low radiologic density with a Hounsfield number of 10 or less within the pelvic cavity, excluding intra-abdominal or pelvic organs. This radiographic parameter served as an objective indicator for the presence of fluid accumulation in the pelvic region. This criterion was used to identify and differentiate the presence of ascites. The ascites volume was estimated with a ruler grid applied using the dimension and cross-sectional thickness of the fluid in the abdominal CT image. The interval between the serial images obtained in our study was 1 cm, and based on this interval, the estimated volumes were calculated. In cases where fluid densities were evident across multiple images, the individual volumes measured on each image were compiled to derive the cumulative volume of ascites. 

## Statistical Analysis

To assess the statistical significance of the observed differences, we employed appropriate statistical tests in our analysis. Specifically, for comparisons between median values, the *t*-test was employed, allowing us to identify any significant variations between the groups being compared. Furthermore, to evaluate the impact of different factors on overall survival (OS), the *χ* ^2^ test was employed, enabling us to determine any notable associations between variables of interest. To further investigate the relationship between the presence of MPF and OS, we utilized Kaplan–Meier analysis. This widely recognized and widely used statistical method allowed us to assess the potential impact of MPF on the survival outcomes of the patients in our study. The OS was defined as the time interval between the initial tissue diagnosis and the occurrence of death from any cause or the last available follow-up for patients whose survival data were censored. To comprehensively assess the prognostic impact of MPF on survival, we performed both univariate and multivariate analyses using Cox proportional hazard models. Through these analyses, we aimed to determine the independent influence of MPF on the survival outcomes of the patients, while accounting for other relevant factors.

## Results

### Patient Demographic Data

**Figure 1.**
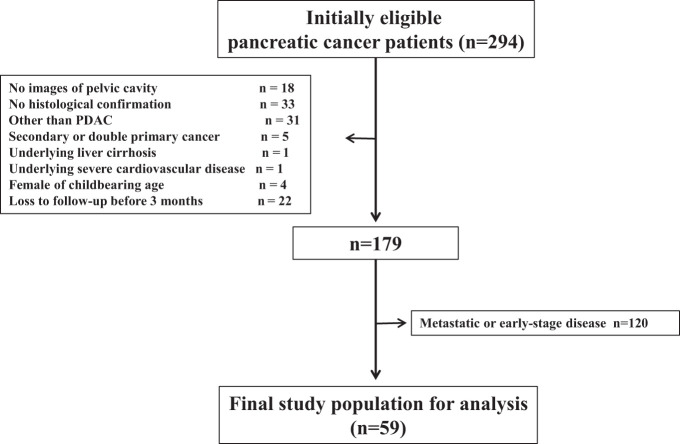
Flowchart of the study population. PDAC, pancreatic ductal adenocarcinoma.

In the initial stage of our study, we conducted a thorough screening of a total of 294 consecutive patients who had received a diagnosis of pancreatic cancer within the specified timeframe of January 2005 to December 2015. To ensure the accuracy and reliability of our analysis, we meticulously applied the predefined exclusion criteria, which led to the exclusion of 115 patients from the study. Subsequently, we focused our analysis on the remaining 179 patients who were diagnosed with LAPC. Among the 179 patients with LAPC, a total of 59 individuals were ultimately enrolled in our study for further analysis, in accordance with our study protocol. These were patients without any evidence of other metastasis. A detailed flow chart illustrating the inclusion and exclusion criteria and patient distribution is provided in Figure 1. Upon examining the demographic characteristics of the enrolled patients, we observed that the median age was 68 years, with 33 patients (55.9%) being male, and both the MPF and no pelvic fluid (NPF) groups had similar median patient ages, with 61.5% (8 out of 13) of patients in the MPF group being male. 

### Patient Clinical Characteristics

Upon examining the baseline clinical characteristics of the 2 groups, we observed no significant differences, except for a higher prevalence of DM in the NPF group, as presented in Table 1. Additionally, although not statistically significant, the CA 19-9 level, primary tumor size, and presence of lymph node metastasis tended to be slightly higher in the MPF group. In terms of the Eastern Cooperative Oncology Group (ECOG) performance status (PS), 13 of the study subjects were classified as ECOG PS 1, while the remaining 46 patients fell under ECOG PS 2. It is worth noting that the distribution of ECOG PSs was relatively similar between the 2 groups, indicating comparable functional statuses among the patients. Among the 59 patients included in our study, a notable finding was that 13 individuals, accounting for 22.0% of the cohort, exhibited the presence of MPF based on the criteria defined in our study protocol. In contrast, the remaining 46 patients (78.0%) showed no evidence of pelvic fluid (NPF).

## Patterns of Treatment

During the period of this study, which spanned from 2005 to 2015, it should be noted that none of the patients received the current standard first-line chemotherapy regimens, such as 5-fluorouracil/leucovorin combined with irinotecan and oxaliplatin (FOLFIRINOX) or gemcitabine–Abraxane. The treatment landscape for LAPC has evolved since then, with the introduction of these more recent therapeutic options. Instead, the majority of patients (86.4%; MPF 92.3%, NPF 84.8%) in our study underwent chemotherapy utilizing a gemcitabine-based regimen. Only 2 patients in the NPF group received concurrent chemoradiation therapy as an upfront treatment presumably because most of the study subjects were at a higher stage and had lymph node involvement, despite no evidence of metastasis. In addition, surgical treatment was attempted as a subsequent treatment in 7 patients (2 patients from the MPF group and 5 patients from the NPF group). Of these patients, 1 underwent R0 resection, while another patient underwent R1 resection. The remaining 5 patients underwent R2 resection exclusively. In the 1 case in which R0 resection was possible, liver metastasis was confirmed immediately after surgery; this patient survived only 5 months. The majority of patients who underwent surgery did not live longer than 6-8 months, and thus, these patients may not have been suitable for surgical treatment.

### Survival Analysis

**Figure 2.**
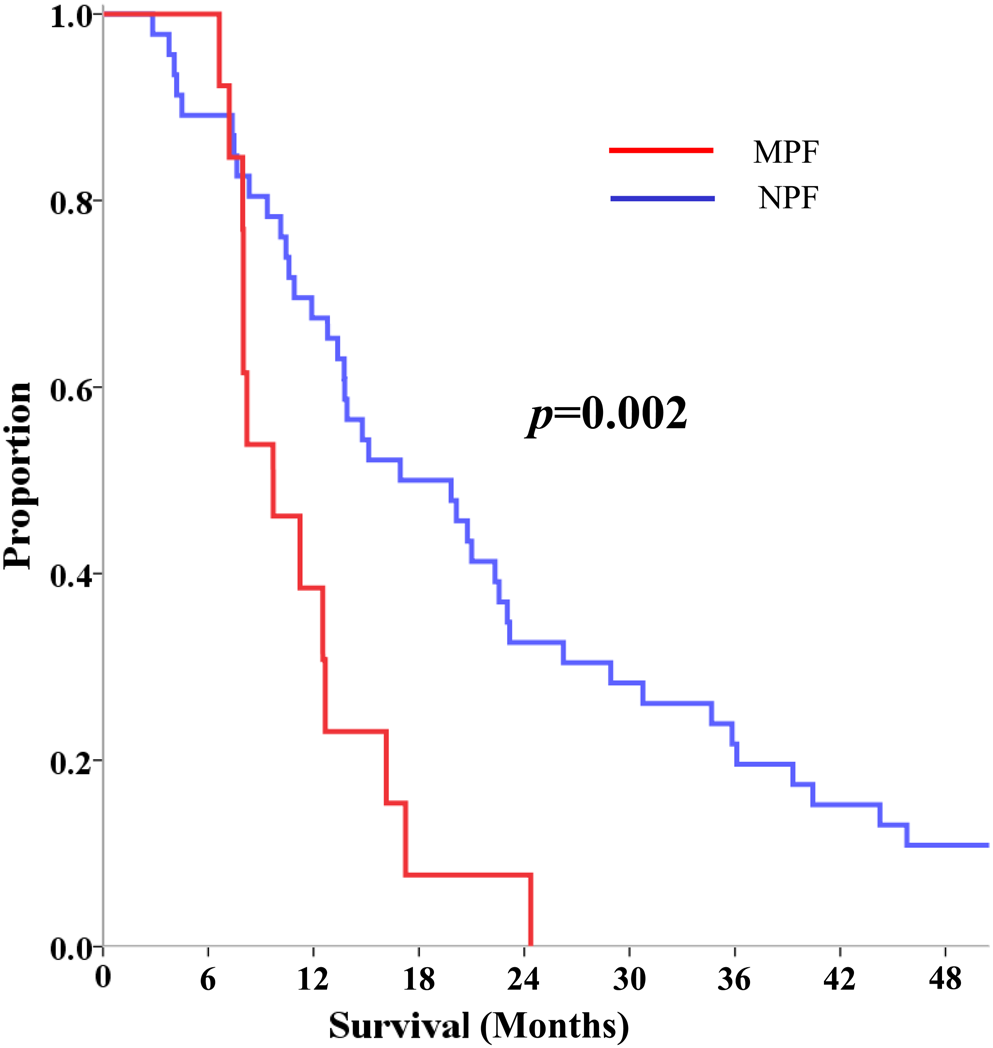
Cumulative overall survival according to the presence (MPF group; red line) or absence (NPF group; blue line) of MPF. MPF, minimal pelvic fluid; NPF, no pelvic fluid.

To assess the impact of MPF on OS in patients diagnosed with PDAC, we employed the Kaplan–Meier method to estimate OS. The results revealed a notable difference in median OS between the MPF and NPF groups. In the MPF group, the median OS was calculated to be 9.7 months, with a 95% CI ranging from 5.9 to 13.5 months. In contrast, the NPF group exhibited a significantly longer median OS of 16.9 months, with a 95% CI spanning from 9.3 to 24.5 months (*P* = .002). These findings, as illustrated in Figure 2, highlight the potential prognostic significance of MPF in the context of PDAC. Among the patients in the MPF group, 2 individuals underwent attempted surgery; however, only palliative surgery could be performed due to the advanced stage of the disease and the extent of tumor involvement. In the NPF group, surgery was attempted in 5 patients, and 3 underwent pylorus-preserving pancreatoduodenectomy. 

To comprehensively explore potential prognostic factors in patients with LAPC, we performed a univariate analysis, considering various variables such as age, ECOG performance status, albumin level, operation status, CA 19-9 level, smoking history, CRP level, and the presence of MPF. Among these factors, our analysis revealed that only the presence of MPF exhibited a significant association with poor prognosis (*P* = .003) in patients with LAPC. This finding underscores the clinical relevance of MPF as an independent predictor of adverse outcomes in LAPC. Furthermore, a multivariate analysis was performed to further investigate the relationship, which revealed a statistically significant association between the presence of MPF and OS (*P* = .01). The comprehensive results of both the univariate and multivariate analyses, including the statistical findings and relevant details, are presented in Table 2.

## Discussion

In this study, median OS in the MPF group [9.7 months, (95% CI, 5.9-13.5)] was obtained to be significantly shorter than that in the NPF group [16.9 months, (95% CI, 9.3-24.5)] (*P* = .002). Furthermore, multivariate and univariate analyses showed that the presence of MPF was an independent poor prognostic factor in LAPC. 

Although MPF have been reported to be associated with a poor prognosis for other gastrointestinal cancers, little data were available on its impact in pancreatic cancer, and the clinical relevance of MPF was unknown. One study on a cohort with metastatic disease reported that patients with ascites had a higher risk of mortality than those without [cHR, 1.63 (95% CI, 1.06-2.51); *P* = .03], but no significant OS difference was detected.^10^ In another study, the authors reported that isolated pelvic metastatic disease rarely occurs in patients with pancreatic cancer and suggested that routine CT follow-up of the pelvic area may be unnecessary. However, tumor stage in the population analyzed was relatively heterogeneous; 153 patients (61.9%) were initially surgically resectable and 94 (38.1%) were locally advanced at diagnosis. Only 1 patient showed isolated metastasis in the pelvic area during follow-up. Furthermore, the authors arbitrarily classified ascites volumes as small, moderate, and large; the standard of classification was not provided, and a small volume of ascites was defined as benign.^18^

In the current study, our inclusion criteria specifically targeted patients with a “minimal” amount of radiographic fluid in the pelvic cavity at the time of diagnosis. By adopting a more stringent definition of MPF, we aimed to concentrate on patients who had a small quantity of ascites, which might otherwise be easily overlooked during the initial staging workup. By concentrating on this specific subgroup of patients, we aimed to shed light on the clinical relevance and prognostic implications of MPF in the context of pancreatic cancer. In a previous study on gastric cancer patients, minimal ascites was defined as volume of <50 mL as determined by abdominal CT, and it was found to be associated with peritoneal carcinomatosis in only 12.5%.^19^ In our study conducted on LAPC patients, we defined MPF as a pelvic fluid volume of less than 100 mL. Remarkably, our study revealed that a considerable proportion, specifically 22%, of patients diagnosed with LAPC exhibited MPF without any discernible evidence of other metastases. However, due to the limited scope of our study, further investigations on a larger scale are warranted to precisely ascertain the true incidence of MPF in the LAPC patient population.

The National Comprehensive Cancer Network Clinical Practice Guidelines in Oncology (NCCN Guidelines) recommend systemic chemotherapy, induction chemotherapy followed by chemoradiation, or stereotactic body radiation therapy in selected patients who are not candidates for combination chemotherapy for the treatment of LAPC. In our study, all patients received systemic chemotherapy as a first-line treatment. The small subset of our patients underwent surgical resection as a sequential treatment, but none of the 13 patients in the MPF group was a candidate for R0 resection. This result suggests that surgical resection does not help to improve the outcomes of patients with MPF, because these patients have similar survival and prognosis with those who have a stage IV metastatic pancreatic cancer. 

In our study, we observed a significant difference between the median survival of patients in the NPF group and the MPF group. The NPF group exhibited a significantly longer median survival (16.9 months) than the MPF group (9.7 months). These findings are consistent with the historically reported median OS ranging from 6.7 to 11.1 months for metastatic pancreatic cancer.^8, 20-22^ Based on these results, it is evident that the presence of MPF should be considered as a poor prognostic factor equivalent to stage IV metastatic pancreatic cancer and that patients with MPF should be regarded as having metastasis. The association of MPF with a worse prognosis in our study raises intriguing questions regarding its underlying mechanisms. While the exact reasons remain uncertain, several factors may contribute to this observed correlation. One possibility is that MPF could serve as an indirect marker of peritoneal carcinomatosis. Another consideration is the potential impact of undetected or subclinical peritoneal metastasis, which may manifest as minimal ascites on imaging. Furthermore, it is important to acknowledge the limitations in accurately characterizing the nature of CT-defined minimal ascites. The challenge lies in distinguishing between exaggerated physiological fluid and true pathological ascites, as well as differentiating between true-positive and false-positive findings. Furthermore, our results also suggest the pelvis should be included when performing abdominal CT scans during initial stage workups in patients with newly diagnosed pancreatic cancer, and that assessments of the presence of MPF are essential for treatment decision-making and the accurate staging of LAPC.

Our study had several limitations. First, the study is limited by its retrospective design; therefore, many unknown confounding factors may have biased our results. However, univariate and multivariate analyses showed that MPF was an independent poor prognostic factor. Second, the definition of minimal ascites as a volume of less than 100 mL may be considered somewhat arbitrary. However, this cutoff value was chosen based on previous studies which were conducted primarily on gastric cancer patients, as there is limited research available specifically on ascites in pancreatic cancer.^19, 23^ These limitations should be taken into account when interpreting the results of our study and considering its implications. Third, despite being a multicenter study the patient number of this study was relatively small. Further analyses targeting larger cohorts may be necessary to validate our findings.

In conclusion, the presence of MPF was found to be significantly associated with poor prognosis in patients with LAPC. Despite the limitations of this study, it is the first multicenter study to evaluate the incidence and clinical significance of MPF in LAPC. Our findings suggest that evaluation of the pelvic area should be included in the initial staging of newly diagnosed pancreatic cancer, due to the association between MPF and poor survival in LAPC patients. Finally, our findings emphasize that MPF in LAPC patients should not be regarded as physiologic fluid.

## Figures and Tables

**Table 1.** Baseline Characteristics of the Patients

	NPF	MPF	P*
	(n = 46)	(n = 13)	
Median age, year^§^	68 (51-81)	68 (46-82)	.98
Sex, male (%)	25 (54.3)	8 (61.5)	.66
DM (%)	14 (30.4)	1 (7.7)	.03
HTN (%)	8 (17.4)	4 (30.8)	.37
Chemotherapy	–	–	.21
Gemcitabine mono (%)	13 (28.3)	2 (15.4)	–
GT (%)	25 (54.3)	6 (46.2)	–
FP (%)	2 (4.3)	1 (7.7)	–
GC (%)	1 (2.2)	1 (7.7)	–
TS-1 (%)	2 (4.3)	0 (0)	–
Others (%)	3 (6.5)	3 (23.1)	–
Radiation therapy (%)	2 (4.3)	0 (0)	.16
CA 19-9^§^	237.45 (0.8-5400.1)	700 (10.73-7612.8)	.12
CRP^§^	0.5 (0.1-71.7)	1.4 (0.1-10.78)	.34
Primary tumor size (cm)^§^	3.7 (2.0-5.4)	4.2 (2.5-5.9)	.15
LN metastasis	44 (95.7)	13 (100)	.16
ECOG PS	–	–	.23
ECOG 1 (%)	8 (17.4)	3 (23.1)	–
ECOG 2 (%)	38 (82.6)	10 (76.9)	–

CA 19-9, carbohydrate antigen 19-9; CRP, C-reactive protein; DM, diabetes mellitus; ECOG, Eastern Cooperative Oncology Group; FP, 5-fluorouracil plus cisplatin; GC, gemcitabine plus capecitabine; GT, gemcitabine plus erlotinib; HTN, hypertension; LN, lymph node; MPF, minimal pelvic fluid; NPF, no pelvic fluid; PS, performance status; TS-1, tegafur, gimeracil, and oteracil potassium.

^§^Median (range).

*P-values were calculated using the *t*-test or the chi-square test.

**Table 2.** Univariate and Multivariate Analyses of Prognostic Factors

Variables	Univariate Analysis		Multivariate Analysis	
	HR (95% CI)	P^*^	HR (95% CI)	P^*^
Age	0.99 (0.98-1.01)	.73	1.01 (0.62-1.41)	.47
ECOG	1.92 (1.57-2.26)	.06	2.22 (1.82-2.63)	.05
Albumin	0.88 (0.78-0.97)	.17	0.89 (0.78-1.01)	.34
Operation	1.84 (1.43-2.25)	.14	1.10 (0.61-1.59)	.85
CA 19-9	1.06 (0.79-1.32)	.12	1.33 (1.04-1.62)	.33
Smoking	1.08 (0.78-1.38)	.80	1.20 (0.85-1.55)	.61
CRP	0.99 (0.98-1.00)	.47	0.98 (0.96-1.00)	.21
MPF	2.75 (2.40-3.09)	.003	2.17 (1.78-2.56)	.01

CA 19-9, carbohydrate antigen 19-9; CRP, C-reactive protein; ECOG, Eastern Cooperative Oncology Group; HR, hazard ratio; MPF, minimal pelvic fluid.

*P-values were calculated using the *t*-test or the chi-square test.
